# ﻿A new species of the genus *Dentatissus* Chen, Zhang & Chang (Hemiptera, Fulgoroidea, Issidae) from the Korean Peninsula, with a key to the genus

**DOI:** 10.3897/zookeys.1173.106206

**Published:** 2023-08-01

**Authors:** Jaekook Park, Sunghoon Jung

**Affiliations:** 1 Laboratory of Systematic Entomology, Department of Applied Biology, College of Agriculture & Life Sciences, Chungnam National University, Daejeon, Republic of Korea Chungnam National University Daejeon Republic of Korea; 2 Department of Smart Agriculture Systems, College of Agriculture & Life Sciences, Chungnam National University, Daejeon, Republic of Korea Chungnam National University Daejeon Republic of Korea

**Keywords:** Auchenorrhyncha, Fulgoromorpha, identification key, Kodaianellini, Korea, pest species, planthopper, taxonomy

## Abstract

A new species of the family Issidae (Hemiptera, Auchenorrhyncha, Fulgoroidea), *Dentatissuslongispinosus***sp. nov.**, is described from the Korean Peninsula. Morphological information is presented with photographs based on male and female specimens. A key to species of the genus *Dentatissus* is also provided.

## ﻿Introduction

The planthopper family Issidae Spinola (Hemiptera, Auchenorrhyncha, Fulgoromorpha) is the fifth-largest group in the superfamily Fulgoroidea, comprising 223 genera and 1,097 species distributed worldwide (Bourgoin 2023). Issidae is a morphologically unique taxon in the superfamily Fulgoroidea, with a generally oval or obovate body shape and with some taxa having a coriaceous forewing (Wang et al. 2016; Gnezdilov et al. 2022). The tribe Kodaianellini Wang, Zhang & Bourgoin belongs to the subfamily Issinae Spinola, which comprises six genera (*Dentatissus* Chen, Zhang & Chang; *Kodaianella* Fennah; *Kodaianellissus* Wang, Bourgoin & Zhang; *Neokodaiana* Yang; *Sivaloka* Distant; *Tetricissus* Wang, Bourgoin & Zhang), which are mainly distributed in East Asia (Wang et al. 2016; Gnezdilov et al. 2022; Bourgoin 2023).

The genus *Dentatissus* Chen, Zhang & Chang belonged to the tribe Issini Spinola, but was transferred to the tribe Kodaianellini Wang, Zhang & Bourgoin (Wang et al. 2016). This group comprises three species [*D.brachys* Chen, Zhang & Chang, *D.damnosus* (Chou & Lu) and *D.quadruplus* Meng, Qin & Wang] and is distinguished by an anal tube with its maximum width near the middle in dorsal view and a phallic complex with two hooked processes (Chen et al. 2014; Chang et al. 2020; Zhang et al. 2020). *Dentatissusdamnosus* is also known as an apple pest in China (Chou et al. 1985; Chen et al. 2014; Wang et al. 2016) (see detailed biology in Chen et al. 2014). In Korea, only *D.brachys* has been recorded up to date (Gnezdilov 2022).

In this study, *D.longispinosus* sp. nov. is described and recorded from the Korean Peninsula. Diagnosis and description of the new taxon along with photographs of the habitus and genitalia, in addition to a key to the species of *Dentatissus*, are provided.

## ﻿Material and methods

### ﻿Specimen collection

Specimens were collected in late August 2021 by sweeping the higher branches and leaves of *Acerpalmatum* near paddy fields. Each individual was immediately aspirated and preserved in 99% ethanol (EtOH).

### ﻿Observation, dissection, and type depository

Photographs of the habitus and measurements were taken using a LEICA DMC2900 adapted with a LEICA M165C microscope and Leica Application Suite Interactive Measurements ver. 4.13. All measurements are given in millimeters (mm). The forewing and hindwing were cut off from the thorax and placed in glycerin for observing veins. Female and male genitalia were carefully extracted using fine needles and subsequently soaked in 10% KOH solution at 70 °C for 10 minutes until cleared, and then placed on glass slides with glycerin for dissection. Illustrations of the male genitalia were created using parchment paper by tracing photographs and rendering the details in freehand while referring to actual specimens. The type specimens are deposited in **CNU** (Laboratory of Systematic Entomology, Chungnam National University, Daejeon, Korea). Distribution and host plant with an asterisk (*) indicate a new record. The distribution map was created using SimpleMappr (Shorthouse 2010).

Morphological terminology follows Bourgoin and Huang (1990), Bourgoin (1993), Gnezdilov et al. (2014) and wing venations as described by Bourgoin et al. (2015).

## ﻿Taxonomy

**Order Hemiptera Linnaeus, 1758**

**Suborder Auchenorrhyncha Duméril, 1806**

**Infraorder Fulgoromorpha Evans, 1946**

**Superfamily Fulgoroidea Latreille, 1807**

**Family Issidae Spinola, 1839**

**Subfamily Issinae Spinola, 1839**

### ﻿Tribe Kodaianellini

Taxon classificationAnimaliaHemipteraIssidae

Wang, Zhang & Bourgoin, 2016

B8A211B5-72BF-5EC2-B31F-E7AA59C0415C

Kodaianellini Wang, Zhang & Bourgoin, 2016: 232.

#### Type genus.

*Kodaianella* Fennah, 1956 (type designated by Wang et al. 2016: 232).

### ﻿Genus Dentatissus

Taxon classificationAnimaliaHemipteraIssidae

Chen, Zhang & Chang, 2014

D35996EF-A54B-5646-B368-8C061F9E171D

Dentatissus Chen, Zhang & Chang, 2014: 140.

#### Type species.

*Dentatissusbrachys* Chen, Zhang & Chang, 2014, by original designation.

#### Diagnosis.

**Figure 1.**
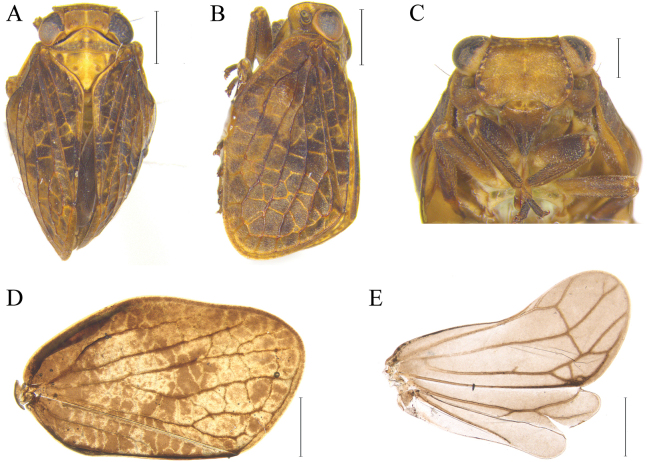
Holotype habitus of *Dentatissuslongispinosus* sp. nov. **A** dorsal view **B** lateral view **C** metope, anteroventral view **D, E** right forewing and hindwing observed in alcohol, respectively. Scale bars: 1.0 mm (**A, B, D, E**); 0.5 mm (**C**).

**Figure 2.**
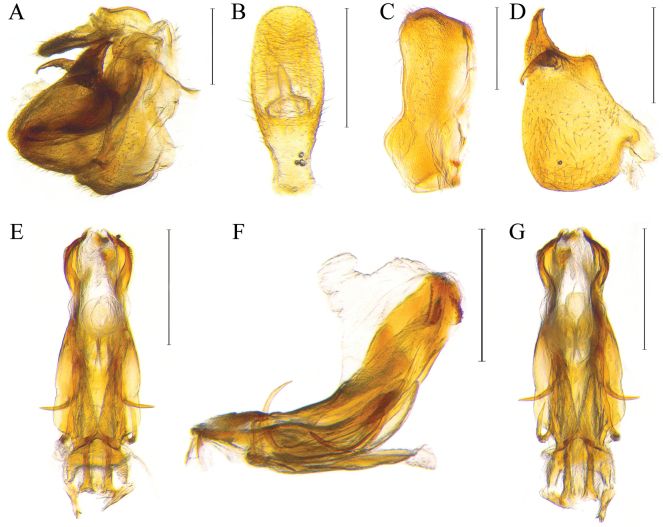
Male genital structures of *Dentatissuslongispinosus* sp. nov. **A** genitalia, lateral view **B** anal tube, dorsal view **C** pygofer, lateral view **D** genital style, lateral view **E–G** phallic complex in dorsal, lateral, and ventral view, respectively. Scale bars: 0.5 mm (**A–G**).

Recognized by general coloration of body brownish to fuscous; coryphe quadrated, not elongated (Fig. 1A, B); anal tube with the maximum width near middle in dorsal view and phallic complex with two ventral aedeagal hooks (Figs 2E–G, 4A–C); genital style with long tooth at base of capitulum (extracted from Chen et al. 2014 and Chang et al. 2020).

#### Distribution.

Korea (South Chungcheong*, South Gyeongsang Province), China (North, Northwest, East, Southwest, South-central regions) (Fig. 5).

### ﻿Key to the species of genus *Dentatissus* Chen, Zhang & Chang

**Table:** 

1	Anal tube concave apically; three or four pairs of aedeagal hooks, phallic complex in lateral view with upper pair of ventral aedeagal hooks relatively short, not surpassing dorsal margins	**2**
–	Anal tube convex apically (Fig. 2B); two pairs of aedeagal hooks, phallic complex in lateral view with upper pair of ventral aedeagal hooks elongated and surpassing dorsal margins (Figs 2F, 4B)	***D.longispinosus* Park & Jung, sp. nov.**
2	Three pairs of aedeagal hooks	**3**
–	Four pairs of aedeagal hooks	***D.quadruplus* Meng, Qin & Wang**
3	Apical portion of male anal tube ovate, as long as middle; anus relatively big; phallic complex in posteroventral view with short ventral aedeagal hooks	***D.brachys* Chen, Zhang & Chang**
–	Apical portion of male anal tube not ovate, narrower than middle; anus small; phallic complex in posteroventral view with elongated ventral aedeagal hooks	***D.damnosus* (Chou & Lu)**

### ﻿ Dentatissus longispinosus

Taxon classificationAnimaliaHemipteraIssidae

Park & Jung sp. nov.

BE33B0A1-8CAF-595E-B273-477665A03D8F

https://zoobank.org/FA853C0F-A597-4560-B1A8-BEE2A5360C1D

**Figure 3.**
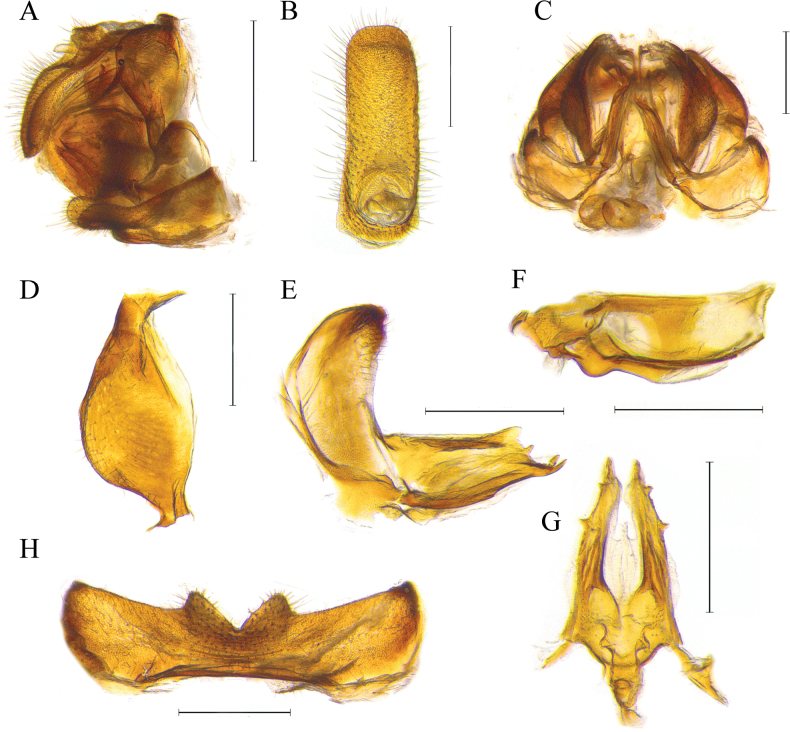
Female genital structures of *Dentatissuslongispinosus* sp. nov. **A** female genitalia, lateral view **B** anal tube, dorsal view **C** female genitalia without sternum VII, caudal view **D** gonoplac, lateral view **E** gonapophysis VIII, lateral view **F** gonapophysis IX, lateral view **G***ditto*, ventral view **H** sternum VII, ventral view. Scale bars: 1.0 mm (**A**); 0.5 mm (**B–H**).

**Figure 4.**
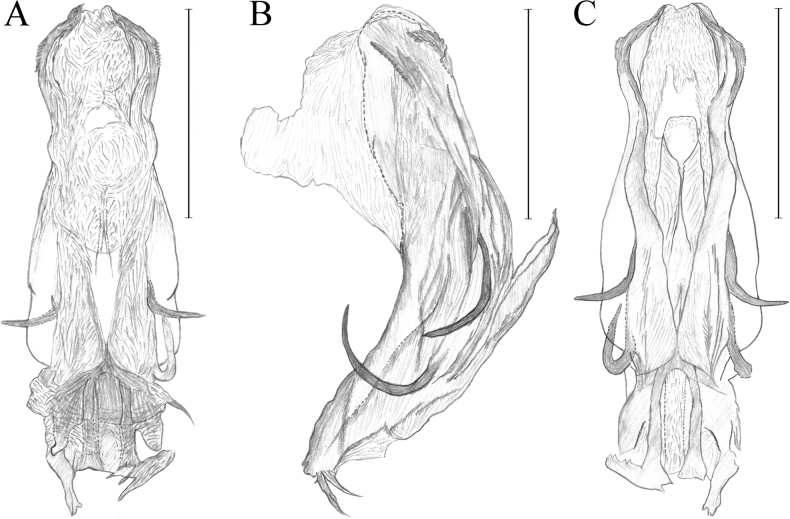
Male phallic complex illustrations of *Dentatissuslongispinosus* sp. nov. **A** dorsal view **B** lateral view **C** ventral view. Scale bars: 0.5 mm (**A–G**).

Figs 1A–E , 2A–G , 3A–G , 4A–C

#### Diagnosis.

Recognized by overall coloration brownish; forewing with fuscous markings developed irregularly, contrasted with background (Fig. 1A, B). Metope without markings, lower half concolorous (Fig. 2C). Phallic complex having two pairs of ventral aedeagal hooks, third pair not developed; upper pair developed horizontally and lower diverging to middle, attached to aedeagus (Figs 2E–G, 4A–C). Anal tube convex apically (Fig. 2B).

#### Description.

**Male. *Coloration*.** General coloration of body brown; Coryphe concolorous with background, median carinae lighter than background; Pronotum yellowish-brown, half to posterior margins darker than background distinctly; mesonotum yellowish-brown, with fuscous markings between median and lateral carinae (Fig. 1A, B); Metope concolorous with background, all margins with yellowish spots irregularly, lateral margins with fuscous and contrast with background; rostrum darker than background. Gena concolorous with background (Fig. 1C). Forewing of veins darker than background, with fuscous markings developed irregularly (Fig. 1A, B). Legs darker with background generally, joints lighter than background (Fig. 1B). Female. Same as male in general features, body relatively larger than male.

***Surface and vestiture*.** Body without wax layer, rough.

***Head*.** Head with compound eyes roundly ovate, larger than pronotum distinctly; Coryphe at base concaved, length shorter than width at midline, apex angulated and slightly protruded after compound eyes, median carinae well-developed (Fig. 1A). Metope ovate, lateral keels protruded and rounded in ventral view, widest at middle, median carina present, margins with postclypeus concaved. Postclypeus and anteclypeus with weak median carina, lateral carinae absent. Antennae short, clavated. Compound eyes semicircular; ocelli absent (Fig. 1C).

***Thorax*.** Pronotum triangular, length longer than width; anterior margins longest at middle, posterior margin slightly concave at midline; median and lateral carinae developed weakly. Mesonotum triangular, length shorter than width, posterior margins pointed; median and lateral carina visually developed (Fig. 1A). Forewing irregularly ovate. Pcu and CuP reaching apical margins, CuA dividing at almost hind margins, Mp and Rp dividing at almost 1/3 (Fig. 1D); Hindwing as long as forewing, trilobed (Fig. 1E).

***Male genitalia*.** Pygofer in lateral view slightly wide basally, margins irregularly (Fig. 2C); Phallic complex long and symmetrical, complex endosome arising widely at apex (Figs 2E, G, 4A, C). Two pairs of ventral aedeagal hooks developed at ventral; the upper pair strongly curved to upward and surpassing aedeagus; the lower curved and not surpassing aedeagus (Figs 2F, 4B); genital style roundly widest at basal, pointed at apical; anterior margins simply protruded at middle, blunt; capitulum of style spinous, curved to ventrally (Fig. 2D); Anal tube in dorsal view, ovate and convex apically; anus placed at middle, anal column small, not reaching the posterior margin (Fig. 2B).

***Female genitalia*.** Gonapophysis VIII partly flattened; dorsal margins almost straight, with sharp apex and well visible teeth at posterodorsal margin (Fig. 3E). Gonapophysis IX in lateral view, slightly curved to dorsally, apical pointed (Fig. 3F); in dorsal view, with two pointed bumps at apical, posterior connective lamina sclerotized (Fig. 3G). Sternum VII with lateral lobes developed, median portion narrow; anterior margins almost straight; posterior margins with two triangular bumps, median portion deeply concaved (Fig. 3H). Anal tube elongated, with numerous setae developed; anus placed basal, anal column short and not surpassing the median portion (Fig. 3B). Gonoplac oval, widest at middle (Fig. 3D).

#### Measurements (mm).

Male (*N* = 2)/Female (*N* = 2). Body length (including forewing): 4.91–4.94/5.53–5.55; body width (including forewing): 2.51–2.53/2.72–2.75.

#### Material examined.

[**CNU**] ***Holotype***: 1♂, 20 Aug 2021, Gung-dong, Yuseong-gu, Daejeon, Republic of Korea. JK Park, sweeping on *Acerpalmatum*; ***Paratype***: 1♂ 2♀♀. Same data as holotype male.

#### Etymology.

This species is named after the elongated processes on the phallic complex.

#### Distribution.

**Figure 5.**
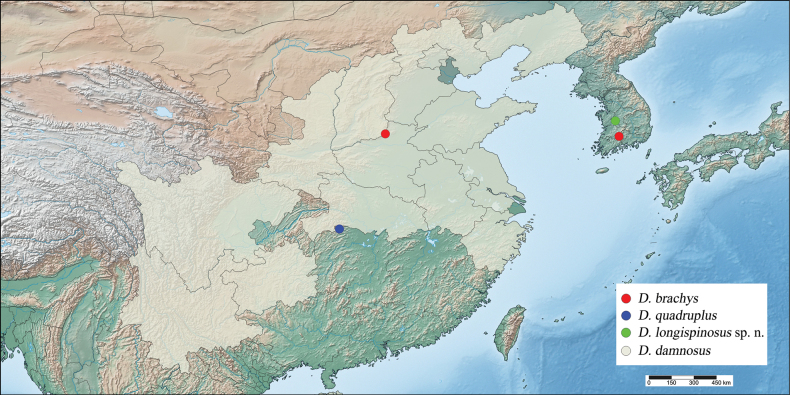
Distribution map of the genus *Dentatissus* species in East Asia.

Korea (South Chungcheong Province) (Fig. 5).

#### Host.

*Acerpalmatum* (Sapindaceae).

## ﻿Discussion

This new species resembles *D.brachys* Chen, Zhang & Chang, 2014 and *D.damnosus* (Chou & Lu, 1985) in general features, but differs in having the anal tube ovate, anus much smaller than other species (Fig. 2B), ventral aedeagal hooks of phallic complex elongated, surpassing dorsal margins (Figs 2E–G, 4A–C), and anal tube and convex apically (Fig. 2B). Chen et al. (2014) provided diagnostic characters for species identification within this genus, with features on the prominent aedeagal hooks. However, the positions of the aedeagal hooks can vary depending on the methods used for specimen dissection. Therefore, additional features such as the shape of the anal tube should be considered for accurate identification. Furthermore, as this genus has been distinguished by male genitalia in previous research, it should be a requirement to examine the female genital characters in future studies.

The genus *Dentatissus* is an endemic taxon found in East Asia, specifically in the Korean Peninsula and China (Chou et al. 1985; Chen et al. 2014; Wang et al. 2016; Gnezdilov 2022; Bourgoin 2023). They are known for containing a pest species in China (e.g., *D.damnosus*: fruit trees and *Ligustrumquihoui*, Chou et al. 1985; Chen et al. 2014; Wang et al. 2016) And, recently *D.brachys* has been recorded in Korea (Gnezdilov 2022). This group is closely associated with shrubs, trees, including fruit trees. Therefore, further research is needed on potential pests, including host information and habitat characteristics of *D.longispinosus* sp. nov. and for the genus and related groups.

## Supplementary Material

XML Treatment for Kodaianellini

XML Treatment for Dentatissus

XML Treatment for Dentatissus longispinosus

## ﻿Additional information

### Author ORCIDs

Jaekook Park https://orcid.org/0000-0001-6965-7199

Sunghoon Jung https://orcid.org/0000-0001-6086-0326

### Data availability

All of the data that support the findings of this study are available in the main text.
